# SARS CoV- 2 vaccination induces antibodies against cardiolipin

**DOI:** 10.1186/s13104-022-06180-3

**Published:** 2022-09-07

**Authors:** George Krashias, Anna Pafiti, Elie Deeba, Christina Christodoulou, Marios Pantzaris, Anastasia Lambrianides

**Affiliations:** 1 Postgraduate School, The Cyprus Institute of Neurology and Genetics, Nicosia, Cyprus; 2 Department of Molecular Virology, The Cyprus Institute of Neurology and Genetics, Nicosia, Cyprus; 3 Neuroimmunology Department, The Cyprus Institute of Neurology and Genetics, Nicosia, Cyprus

**Keywords:** COVID-19, Thrombosis, Coagulopathies, ChAdOx1-S, BNT162b2

## Abstract

**Objective:**

Cases of thrombosis have been reported after administration of SARS-CoV-2 vaccines, with controversial results relating to Oxford-AstraZeneca’s ChAdOx1-S. Despite such cases being rare, they still raised concerns for their involvement in coagulopathies. Anti-cardiolipin (aCL) IgG antibodies have been linked to venous and arterial thrombosis. The aim was to evaluate the concentration of aCL IgG antibodies in vaccinated and COVID-19 positive individuals using indirect ELISA and commercial sourced calibrators.

**Results:**

The concentration of aCL IgG antibodies was measured in the serum of COVID-19 positive (n = 37), ChAdOx1-S vaccinated (n = 37) and BioNTech Pfizer BNT162b2 vaccinated (n = 42) individuals. Samples from COVID-19 negative, unvaccinated individuals (n = 41) served as controls. The highest percentage of positivity was in the COVID-19 positive group (18.9%). Concerning vaccination, BNT162b2 had the highest percentage of positivity (11.9%) *(p* = 0.0037). Additionally, aCL concentrations were evaluated at different time points in both vaccinated groups (before, 3 weeks after and 3 months after the second dose). A significant difference in the levels of aCL IgG antibodies over time (*p* = 0.0391) was observed only in ChAdOx1-S individuals. Our study concluded that levels of aCL, after vaccination with either of the vaccines or following SARS-CoV-2 infection, were not clinically pathogenic for the risk of thrombosis.

## Introduction

The spread of Severe Acute Respiratory Syndrome Coronavirus 2 (SARS-CoV-2), a respiratory viral infection, was declared a pandemic by the World Health Organization (WHO) in early 2020 [1]. The new generation of vaccines for Coronavirus 2019 (COVID-19), with the first one making an appearance just a year after the first case of COVID-19, include BioNTech Pfizer’s BNT162b2 and Moderna’s mRNA-1273, which are mRNA-based technology vaccines, act through directly producing an antigen, the spike protein. Their immunogenicity includes high neutralizing antibodies and CD4^+^ and CD8^+^ T cell response [2]. Oxford-AstraZeneca’s ChAdOx1-S and Janssen’s JNJ-78436735 use adenoviral vector-based technology, which are genetically engineered to include the target gene [1]. ChAdOx1-S was created using a Chimpanzee adenovirus vector that displays the spike protein on its surface, developing a strong immune response through high neutralizing antibodies. Janssen’s JNJ-78436735 includes adenovirus serotype 26 that also expresses the spike protein. [2]. Despite vaccines being the best approach, there has been controversy regarding an association between immune thrombocytopenia and some formulations of COVID-19 vaccine. Very rare cases of thrombosis at unusual sites have been reported, mostly associated with thrombocytopenia, shortly after the administration of ChAdOx1-S [3]. In fact, ChAdOx1-S vaccine was temporarily banned in some European countries following a few cases of thrombosis, the formation of blood clots in blood vessels that prevent blood flow [3]. Specifically, anti-cardiolipin (aCL) IgG antibodies have been found to be associated with thrombosis [4]. Detection of aCL IgG antibodies can be a valuable laboratory indicator for thrombotic events [5] or a possible thrombotic condition associated with COVID-19 vaccination.

We aimed to determine the concentration of aCL antibodies in subjects following vaccination with ChAdOx1-S or BNT162b2. Additionally, aCL antibodies were also assessed in individuals positive for COVID-19 using a reverse transcription polymerase chain reaction (RT-PCR) test. All study groups were compared to a control group of non-vaccinated and COVID-19 negative individuals with the purpose of identifying clues as to possible coagulopathies in COVID‐19 vaccination.

## Main text

## Methods

### Study design and participants

Blood samples were collected from 37 SARS-CoV-2- positive individuals, 37 individuals vaccinated with ChAdOx1-S and 42 with BNT162b2. Participants were recruited at the time the vaccination campaign started. February 2021 for BNT162b2 and April 2021 for ChAdOx1-S. Samples were taken at around 3 to 4 weeks after the administration of the second dose. Additionally, samples were collected from 41 individuals that tested negative against SARS-CoV-2 with no history of vaccination. All study participants signed an informed consent form approved by the Cyprus National Bioethics Committee (ΕΕΒΚ/ΕΠ/2020/23).

### Blood processing

Blood samples were collected in BD Vacutainer red-top plain tubes containing clotting activators at The Cyprus Institute of Neurology and Genetics. Samples were allowed to clot at room temperature and centrifuged at 1500 g for 10 min. Serum was extracted and stored at −20 °C.

### aCL antibody detection using an indirect ELISA

aCL IgG antibodies were measured using an indirect ELISA along with commercially sourced calibrators (Louisville APL Diagnostics, USA). In brief, 96-well Polysorp plates were coated with 50 μg/ml cardiolipin (Sigma) in ethanol. Plates were incubated at 4˚C overnight and blocked using 10% HI FBS/PBS for one hour at 37 °C. Serum samples where diluted 1:50 with 10% HI FBS/PBS in triplicate and incubated for 90 min at 37 °C. Bound IgG was detected by the addition of anti-human IgG horseradish peroxidase conjugate, diluted with 10% HI FBS/PBS and incubated for 1 h at 37 °C. 3,3′,5,5′-Tetramethylbenzidine (TMB) Substrate was then added, and absorbance was measured at 450 nm. A linear graph of the concentration of the calibrators against the absorbance measured was created. The graph equation was used to calculate the concentration of the samples. Activity was defined in IgG phospholipid units (GPLU) where 20 GPLU and above is considered positive for the presence of aCL antibodies according to the manufacturer’s instructions. Inter and intra plate variations were assessed according to the protocol of the Louisville APL diagnostics. A 15% allowable difference in the calibrators between each plate does not cause a statistical bias.

### Statistical analysis

All statistical analysis was performed using GraphPad Prism Version 8 for Windows, La Jolla, California, USA. Wilcoxon- Mann–Whitney test and Friedman test were the statistical tests used for the analysis of aCL concentrations at different time points for the two vaccinated groups. Unpaired t-tests were used for the analysis of aCL concentrations at ≈ 21 days after the second dose.

## Results

### Patient demographics

Thirty-seven individuals that were found SARS-CoV-2- positive through RT-PCR were asked to provide blood samples. Similarly, volunteers who were vaccinated with BNT162b2 (n = 42) gave blood samples 3 weeks after the first vaccination dose and those vaccinated with ChAdOx1-S (n = 37) gave blood samples 12 weeks after the first vaccination dose, and repeated sampling was received from both groups ≈ 21 days and 3 months after the second vaccination dose. The study groups were sex and age matched to a control group negative for SARS-CoV-2, not vaccinated by any of the available FDA approved SARS-CoV-2 vaccines. Demographics of the study groups are shown in Table 1.

**Table 1:** Patient Demographics

	Control group	COVID-19 Positive	BNT162b2	ChAdOx1-S
No. of subjects	41	37	42	37
Sex (M/F)	19/22	20/17	20/22	17/20
Mean age (± SD)	47.17 (± 11.01)	45.49 (± 14.06) *P* = 0.555*	46.83 (± 12.64) *P* = 0.897*	44.73 (± 12.98) *P* = 0.379*

*M* males, *F* females, *S.D* standard deviation

P values indicate no significant difference in the ages of all the participants in comparison to the control group

### aCL concentrations at ≈ 21 days after the second dose

**Fig. 1:**
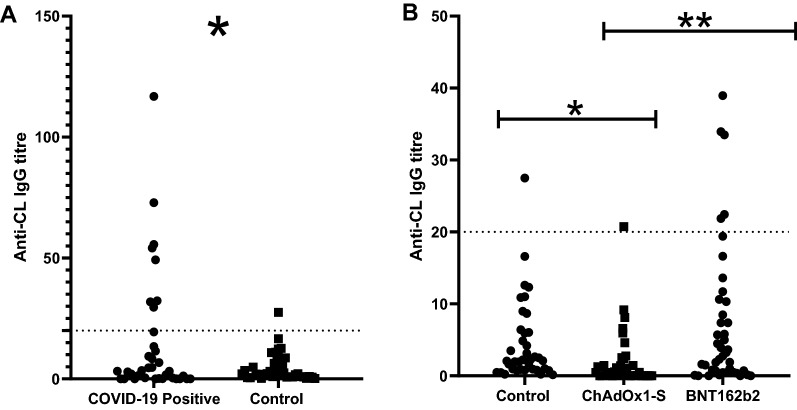
**A** aCL concentrations in COVID-19 positive and controls (COVID-19 negative non-vaccinated individuals). Dashed line indicates the threshold above which samples are considered positive (> 20GPLU). The threshold was determined by the manufacturer's protocol of the calibrators used. **A** significant difference of *p* = 0.0167 was found between the two groups. **B** aCL concentrations of the study groups, vaccinated with BNT162b2 and ChAdOx1-S. The control group is the unvaccinated COVID-19 negative participants. Dashed line indicates the threshold above which the samples are considered positive (> 20GPLU). aCL concentrations between ChAdOx1-S vaccinated and the control group was found to be significantly different *p* = 0.0289. No significant difference (*p* = 0.1414) was found between the BNT162b2 vaccine group and the control. A significant difference (*p* = 0.0037) was found between the two vaccine groups

Overall, 18.9% of SARS-CoV-2- positive individuals tested positive, 11.9% of BNT162b2- vaccinated, 2.7% of ChAdOx1-S- vaccinated and in comparison, 7.3% of the control, SARS-CoV-2 negative non-vaccinated group (Fig. 1). A significant difference (*p* = 0.0289) has been found in the aCL concentration of the ChAdOx1-S- vaccinated in comparison to the control group, with the vaccinated group averaging lower concentrations (Mean = 2.012 GPLU). Similarly, SARS-CoV-2- positive individuals had a significantly (*p* = 0.0167) higher concentration, than the control group (Mean = 4.531 GPLU). No significant difference (*p* = 0.1414) was found between the BNT162b2- vaccinated and the control group. Lastly, when comparing the two vaccines, BNT162b2 had induced significantly (*p* = 0,0037) higher concentrations of aCL.

### Change in aCL IgG antibody concentration at different time points around the second dose of the vaccine

**Fig. 2:**
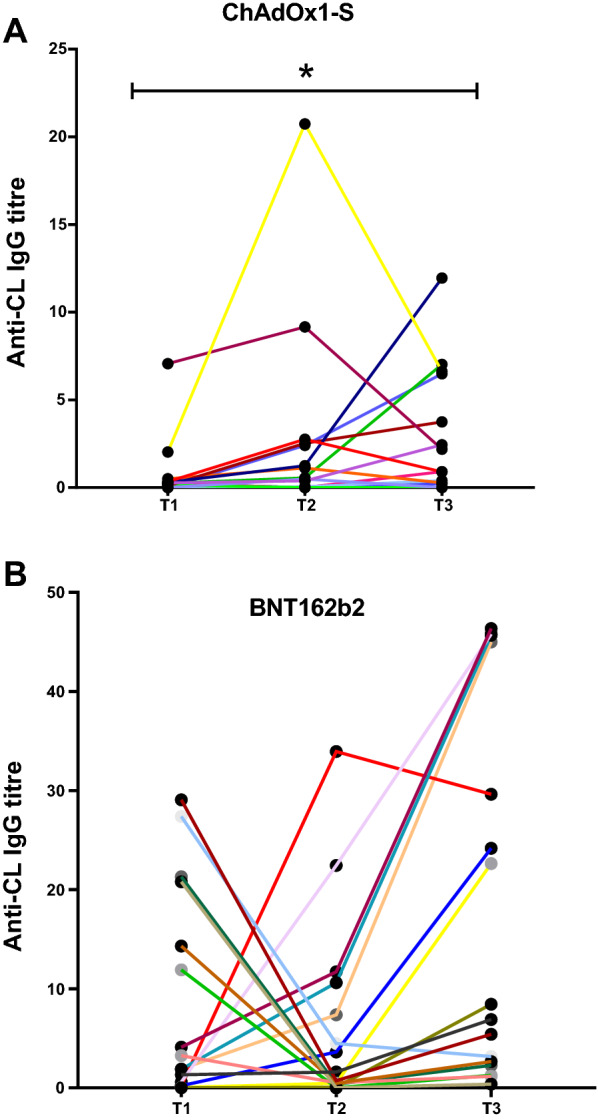
aCL concentration in individuals vaccinated with ChAdOx1-S (**A**) and with BNT162b2 (**B**) across three sampling points, no earlier than a week before the second dose (T1), ≈ 21 days after the second dose (T2) and 3 months after (T3). A significant difference (*p* = 0.00391) was found in the concentration over time for the ChAdOx1-S (Fig. 2A). While no significant difference (*p* = 0.0874) was in the BNT162b2 vaccinated group over time

To determine whether there was a change in the concentration aCL IgG before and after the second dose, additional samples were tested from the same individuals (Fig. 2). No significant difference (*p* = 0.0874) was found between the three samples, before second dose (T1) where IgG concentration measured 2.581 GPLU [Interquartile range 0.308 to 19.18], ≈21 days after second dose (T2) 1.169 GPLU [0.398 to 9.805] and 3 months after (T3) 7.669 GPLU [2.359 to 41.16] of the BNT162b2- vaccinated study group. A significant difference (*p* = 0.0391) was found for the ChAdOx1-S- vaccinated group, where aCL IgG concentration measured 0.2340 GPL [0.078 to 0.3793] at T1, 0.8340 GPLU [0.035 to 2.583] at T2 and 1.543 GPLU [0.255 to 6.523] at T3.

## Discussion

Production of antiphospholipid antibodies is common after viral infections; however, their clinical significance is still unknown. Some patients may have these antibodies for short period of time, yet in some cases they persist and can contribute to the development of autoimmune diseases [6]. aCL is an antiphospholipid antibody, found at high concentrations in the antiphospholipid syndrome (APS). In fact, the hallmark of APS involves the setting of aCL, at the site of thrombosis. Furthermore, it has been found that antibodies induced by SARS-CoV-2 cross-react with red blood cells, platelets and serum albumin which results in blood thickening. Specifically, SARS‐CoV‐2 spike protein is structurally similar enough to some blood coagulation factors [7]. Several studies investigated the safety of the ChAdOx1 vaccine indicating a shared pathophysiological basis of a syndrome similar to autoimmune heparin-induced thrombocytopenia in some individuals [8, 9].

In the present study, aCL IgG antibodies were evaluated in COVID-19 positive patients and individuals vaccinated with BNT162b2 and ChAdOx1-S. All study groups were compared to COVID-19 negative, non-vaccinated individuals. The highest percentage of subjects positive for aCL IgG antibodies was the COVID-19 positive group (18.2%), significantly different (*p* = 0.0167) from the control group. This is in line with a recent study, showing severe cases of COVID-19 are more prone to thrombotic events, with patients admitted to ICU presenting high concentration of aCL IgG antibodies [10, 11]. However, none of the COVID-19 positive subjects included in our study required hospitalization.

Interestingly, BNT162b2 vaccinated group had a higher percentage of positivity for aCL, at 11.9%, in comparison with ChAdOx1-S, at 2.7%, which was also found to be significantly different (*p* = 0.0037). An association between ChAdOx1-S vaccination and thrombosis is reported [3]. Interestingly, individuals vaccinated with ChAdOx1-S had the lowest positivity (2.7%) but also the lowest overall concentration of aCL IgG antibodies following administration of the second dose, even when compared to the non-vaccinated individuals (*p* = 0.0289) (Fig. 1B). Importantly, literature thus far have not explored the full set of groups included in the present study, therefore there is insufficient knowledge in interpreting the presence of aCL antibodies.

Moreover, a significant difference (*p* = 0.0391) was observed between concentration levels of aCL IgG antibodies over time in the study groups vaccinated with ChAdOx1-S (Fig. 2A). On the contrary, a significant difference was not observed in the BNT162b2 vaccinated group. Noteworthy, there is a significant difference after the second dose is administrated for both vaccines. For BNT162b2, T2 samples were around 3 weeks after administration of the first dose, whereas for ChAdOx1-S the second dose was given at around 8 to 12 weeks after the first dose. Assuming both doses spike the exact same response regarding the production of aCL IgG antibodies, ChAdOx1-S vaccinated group had more time to lower levels back to baseline in comparison to BNT162b2. This could also explain the higher concentration and degree of positivity in the BNT162b2 vaccinated group. T2 samples could account for the increase of aCL in both doses together. In addition, this could also explain why the ChAdOx1-S vaccinated group had a significantly lower (*p* = 0.0289*)* concentration that the control group at the T2 time point. Another important detail that needs to be taken into consideration, is the fact that ChAdOx1-S and BNT162b2 differ in the molecular design delivery system used. In the case of ChAdOx1-S, viral vectors are designed to specifically express antigens of target pathogens. The delivery of the target antigen by viral vectors produces potent antigens consequently mimicking the natural infection which then induces strong T cell responses [2]. The chimpanzee adenovirus vector was used due to the low human prevalence, as humans have low pre-existing immunity to its viral backbone [2]. The BNT16b2 vaccine employs the use of S antigen-encoding mRNA. They need to be delivered to the cytoplasm of host cells for translation to occur [2]. Further studies are required, which may provide clues as to the origins of coagulopathies in COVID‐19 and determine whether is due to the production of aCL IgG antibodies.

Noteworthy, a recent study has concluded that antiphospholipid antibodies produced in APS differ from those seen in SARS-CoV-2 [12]. Despite that, it does not mean that aCL linked to SARS-CoV-2, have no association with thrombosis [13, 14]. Severe cases of COVID-19 infection can still benefit from antiphospholipid antibody tests as they can demonstrate the risk of thrombosis in patients and preventative care can be applied.

## Conclusion

To our knowledge, this is the first study that compares levels of aCL IgG antibodies in vaccinated and COVID-19 positive individuals. An anti-phospholipid antibody test may be useful to prevent serious cases of coagulation following vaccination enrolment. Health care providers should be aware of clinical presentation and management strategies associated with post-COVID-19 vaccine-induced thrombotic thrombocytopenia. Our study has shown that aCL concentrations are not increased to the point where they can be considered pathogenic, even at 3 months after vaccination. However, it is vital to know that aCL is not an independent variable of thrombosis and further testing will be required if a patient presents signs of thrombosis [15, 16].

## Limitations

A major limitation of our study is the number of participants and sampling pre-vaccination. We were not able to normalise our results to account for such variations. We also acknowledge the fact that cardiolipin is a normal blood component and additionally the use of ELISA for the detection of anti-CL includes anti-β2-glycoprotein I [17]. We accounted for that by using FCS, which includes β2-glycoprotein I, to block any non-specific binding. Lastly, we are unaware of any coagulopathies our participants could have.

## Data Availability

The raw data of this study are available upon reasonable request to the corresponding author.

## Abbreviations

aCL: Anti-cardiolipin
SARs-CoV-2: Severe acute respiratory syndrome coronavirus 2
COVID-19: Coronavirus 2019
RT-PCR: Reverse transcription polymerase chain reaction
APS: Antiphospholipid syndrome
